# Regulatory T cell therapy suppresses inflammation of oral mucosa

**DOI:** 10.3389/fimmu.2022.1009742

**Published:** 2022-10-31

**Authors:** Ningning Xue, Ying Wang, Hao Cheng, Hantian Liang, Xinzou Fan, Fengqiong Zuo, Xin Zeng, Ning Ji, Qianming Chen

**Affiliations:** ^1^ State Key Laboratory of Oral Diseases, National Clinical Research Center for Oral Diseases, Chinese Academy of Medical Sciences Research Unit of Oral Carcinogenesis and Management, West China Hospital of Stomatology, Sichuan University, Chengdu, Sichuan, China; ^2^ Department of Biotherapy, State Key Laboratory of Biotherapy and Cancer Center, West China Hospital, Sichuan University, Chengdu, Sichuan, China; ^3^ Department of Immunology, West China School of Basic Medical Sciences and Forensic Medicine, Sichuan University, Chengdu, Sichuan, China

**Keywords:** oral mucosa, Treg cells, oral inflammation, CD25, Foxp3, immunotherapy

## Abstract

Oral inflammatory diseases, including oral lichen planus (OLP) and recurrent aphthous ulcer (RAU), seriously affect the patient’s quality of life. Due to the lack of ideal disease models, it is difficult to determine whether novel immunotherapy strategies are effective in treating oral inflammatory diseases. Here, we show that the deficiency of Foxp3 or IL-2 caused oral mucosa inflammation in mice, proving that Treg cells are important in maintaining the immune homeostasis in the oral mucosa. Then we determined that adoptive transfer of CD4^+^CD25^-^CD45Rb^high^ T cells could induce oral inflammation in *Rag1* ^-/-^ mice, and co-transfer of Treg cells together with CD4^+^CD25^-^CD45Rb^high^ T cells could suppress the development of oral inflammation in this mouse model. Our study showed that adoptive transfer of CD4^+^CD25^-^CD45Rb^high^ T cells into *Rag1* ^-/-^ mice could be a novel disease model of oral inflammation. Our data provides direct evidence that Treg cell therapy is effective in suppressing oral mucosa inflammation in mice. Therefore, Treg cell therapy may be a promising novel strategy to treat oral inflammatory diseases.

## Introduction

The majority of oral mucosal diseases are inflammatory diseases (1). Inflammatory diseases of the oral mucosa, including oral lichen planus (OLP) and recurrent aphthous ulcer (RAU) have high prevalence and incidence (2, 3). These diseases either exhibit a long disease course or relapse easily, and also seriously affects the patient’s quality of life. Therefore, the prevalence of depression, anxiety, and stress is high in patients with oral inflammatory diseases (4, 5). Although the pathogenesis of these diseases is largely unknown, an increase in various pro-inflammatory cells and cytokines has been shown to be involved in the development and perpetuation of these diseases (6–10). However, the classic immunosuppressive drugs used to treat these patients have quite limited efficacy and may cause many side effects including anxiety, swelling, and weight gain (11). Therefore, the investigation of novel effective strategies to treat inflammatory diseases of the oral mucosa will benefit patients greatly.

CD4^+^CD25^+^Foxp3^+^ regulatory T cells (Treg cells) are a subset of CD4+ T cells that co-express interleukin-2 receptor alpha chain (IL-2Rα, also called CD25) and the transcription factor forkhead box P3 (Foxp3) (12–15). These Treg cells are the key immune cells in maintaining immune tolerance and suppressing inflammation (16). Manipulating Treg cells has been shown to be a promising strategy to suppress and treat autoimmune and inflammatory diseases, including inflammatory bowel disease (IBD), experimental autoimmune encephalomyelitis (EAE), Type I diabetes (T1D), asthma, and arthritis (17–19). The IL-2-CD25 signaling pathway has been determined to be dramatically important for Treg cell generation in the thymus and periphery, as the absence of IL-2-CD25 signaling pathway cause Treg cell deficiency and severe systemic inflammation (20–22).

A number of studies have reported that Treg cells were increased in the sub-epithelial lymphocytic infiltrate of the OLP lesions and in peripheral blood mononuclear cells (PBMCs) (23–26). Our previous study showed that Treg cell frequency was negatively correlated with immune cell activation in OLP lesions (17). These findings suggest that an enhanced Treg cell function or increased Treg cell frequency can suppress oral mucosa inflammation, such as OLP. However, these studies were based only on phenotypic descriptions and speculations, and none of them determined whether Treg cell therapy could treat inflammatory diseases of the oral mucosa. One major difficulty in determining the function of Treg cells in oral mucosal inflammation is that there are no ideal inflammatory disease models of the oral mucosa, making it difficult to perform rigorous pre-clinical studies to determine whether Treg cell therapy is effective in treating inflammation of the oral mucosa. Therefore, it is necessary to develop ideal inflammatory disease models of the oral mucosa and to determine the therapeutic function of Treg cells in oral mucosal inflammation.

Here, we determined that the deletion of IL-2-CD25 signaling causes inflammation of the oral mucosa in mice. Following these findings, we developed an inflammatory disease model of the oral mucosa by adoptively transferring CD4^+^CD25^-^CD45Rb^high^ T cells into Rag1^-/-^ mice, and determined that Treg cell therapy suppressed inflammation of the oral mucosa.

## Methods and materials

### Mice

C57BL/6 mice were bred in the animal facility of West China Hospital of Stomatology, Sichuan University under specific pathogen-free (SPF) conditions. *Il2^-/-^* mice, Scurfy mice (*Foxp3* ^-/-^ mice), *Rag1^-/-^* mice, Foxp3-eGFP mice (C57BL/6 background), and CD45.1 (C57BL/6 background) mice were obtained from The Jackson Laboratory and bred in the animal facility of Sichuan University under specific-pathogen-free (SPF) conditions. All the mice used in the experiments were aged 6–8 weeks. All animal studies were approved on February 26, 2016, by the Animal Care and Use Committees of the West China Hospital of Stomatology, Sichuan University.

### Antibodies and reagents

Fluorochrome-conjugated anti-mouse CD45.1 (A20), anti-mouse CD45.2 (104), anti-mouse TCR-β (H57-597), anti-mouse CD4 (RM4-5), anti-mouse CD8α (53-6.7), anti-mouse IFN-γ (XMG1.2), anti-mouse TNF-α (MP6-XT22), anti-mouse IL-17A (eBio17B7), and anti-mouse/rat Foxp3 (FJK-16a) antibodies were obtained from Thermo Fisher Scientific. Foxp3/Transcription Factor Staining Buffer Set (00-5523-00) was obtained from Thermo Fisher Scientific. The BD Golgi-Plug Protein Transport Inhibitor (555029) and BD Cytofix/Cytoperm Fixation/Permeabilization Solution Kit (554714) were purchased from BD bioscience. PMA (P8319) and ionomycin calcium salt (I3909) were purchased from Millipore Sigma.

### T cell adoptive transfer model

The CD4^+^CD25^-^CD45RB^hi^ T cell adoptive transfer model was established as previously described (27, 28), with some modifications. CD4^+^CD25^-^CD45RB^hi^ T cells were sorted with flow cytometry (FCM) from the spleens and peripheral lymph nodes (PLNs) of CD45.1 mice; then injected these sorted cells into *Rag1^-/-^* mice intravenously (0.4 × 10^6^ cells per mouse). *Rag1^-/-^* mice received CD4^+^CD25^-^CD45RB^hi^ T cells would develop oral mucosa inflammation 8-10 weeks post T cell transfer. In the Treg cell therapy model, CD4^+^CD25^+^Foxp3(eGFP)^+^ Treg cells (0.1 × 10^6^ cells per mouse) sorted with flow cytometry from spleens and peripheral lymph nodes (PLNs) of Foxp3-eGFP mice were co-transferred with CD4^+^CD25^-^CD45RB^hi^ T cells (0.4 × 10^6^ cells per mouse) into *Rag1^-/-^* mice intravenously to investigate the suppression of oral inflammation.

### Flow cytometry analysis

Spleens and lymph nodes were mashed with 40μm filters to get single-cell suspensions. The cell surface staining was performed by staining the cell surface markers with antibody solutions for 20 minutes at 4°C in the dark. The intracellular cytokine staining was performed by culturing the cells with PMA (5 ng/mL), ionomycin (1μM) and Golgi-Plug (1: 1,000 dilutions) at 37°C for 4 hrs. The cultured samples were then stained with cell surface markers (antibody concentration: 1μg/ml), fixed with BD Fixation/Permeabilization buffer solution and stained with antibody solutions (antibody concentration: 2μg/ml) for 40 minutes at 4°C in the dark. The intranuclear transcription factor staining was performed by staining the cells with cell surface markers (antibody concentration: 1μg/ml), permeabilizing with Foxp3/Transcription Factor Staining Buffer Set, and staining with antibody solutions (antibody concentration: 2μg/ml) for 60 minutes at 4°C in the dark. The gating strategies for the flow data are presented in **Supplementary Figure 1**.

### Statistical analysis

Statistical analysis was performed using unpaired two-tailed Student’s *t*-tests to compare differences between two different groups. Statistical significance was set at *p* < 0.05.

## Results

### Treg cell deficiency causes severe inflammation in oral mucosa

**Figure 1:**
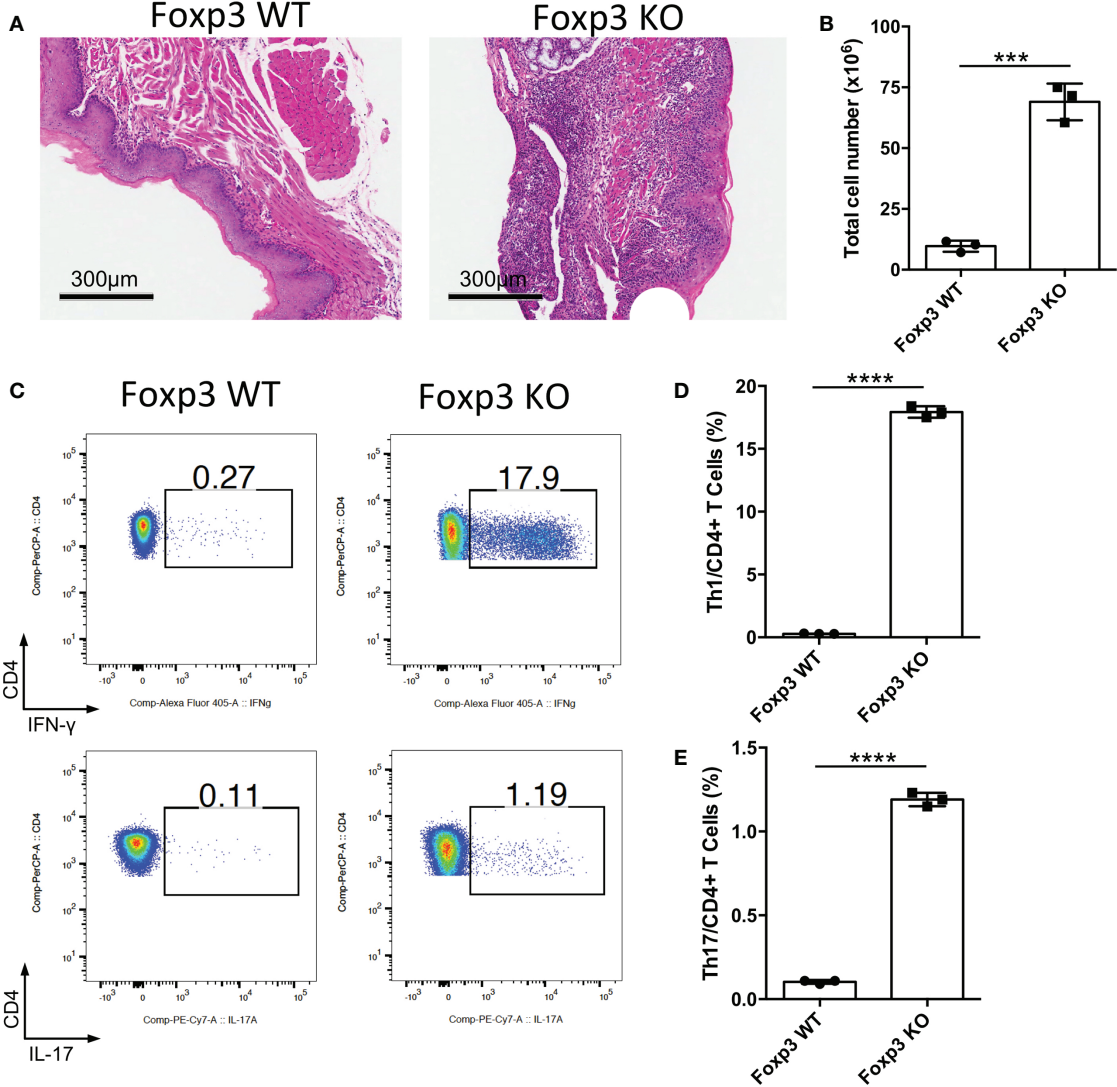
Deletion of Treg cells causes severe inflammation in oral mucosa. Oral tissues and draining lymph nodes (DLNs) of *Foxp3* ^+/+^ (Foxp3 WT) and *Foxp3* ^-/-^ (Foxp3 KO) mice were harvested from four-week-old mice (n=3). **(A)** Representative histology images of oral mucosa sections. Scale bars, 300μm. **(B)** Bar graphs showing total cell numbers in DLNs of the oral cavity. **(C-E)** Representative flow cytometry plots **(C)** and bar graphs **(D, E)** showing frequencies of CD4^+^ IFN-γ^+^ T cells (Th1 cells) and CD4^+^ IL-17A^+^ T cells (Th17 cells) in DLNs. Data are representative of two independent experiments. Summary data are presented as mean ± SD. ***p < 0.001; ****p < 0.0001, unpaired two-tailed Student’s *t*-tests.

Foxp3 is the key transcription factor of Treg cells, and loss of Foxp3 cause the deficiency of Treg cells (13–15). To determine the function of Treg cells in the oral mucosa, we bred *Foxp3* ^-/-^ mice to investigate inflammation development of oral mucosa in these mice. From the histological slides of the oral mucosa of *Foxp3* ^+/+^ and *Foxp3* ^-/-^ mice, we found that the loss of Treg cells resulted in severe inflammation in the oral mucosa (**Figure 1A**). Consistent with this, the total cell number of DLNs in *Foxp3^-/-^ mice* was significantly increased (**Figure 1B**). To check the immune responses of oral cavity, we investigated the frequencies of IFN-γ producing T cells (Th1 cells) and IL-17 producing T cells (Th17 cells) in the cervical lymph nodes (CLNs), the draining lymph nodes (DLNs) of oral cavity. This revealed that the deletion of Treg cells caused a dramatic increase in Th1 cells and Th17 cells (**Figures 1C–E**). These data show that Treg cells are important for maintaining the immune homeostasis in the oral mucosa.

### IL-2-CD25 signaling pathway deficiency causes severe inflammation in the oral mucosa

**Figure 2:**
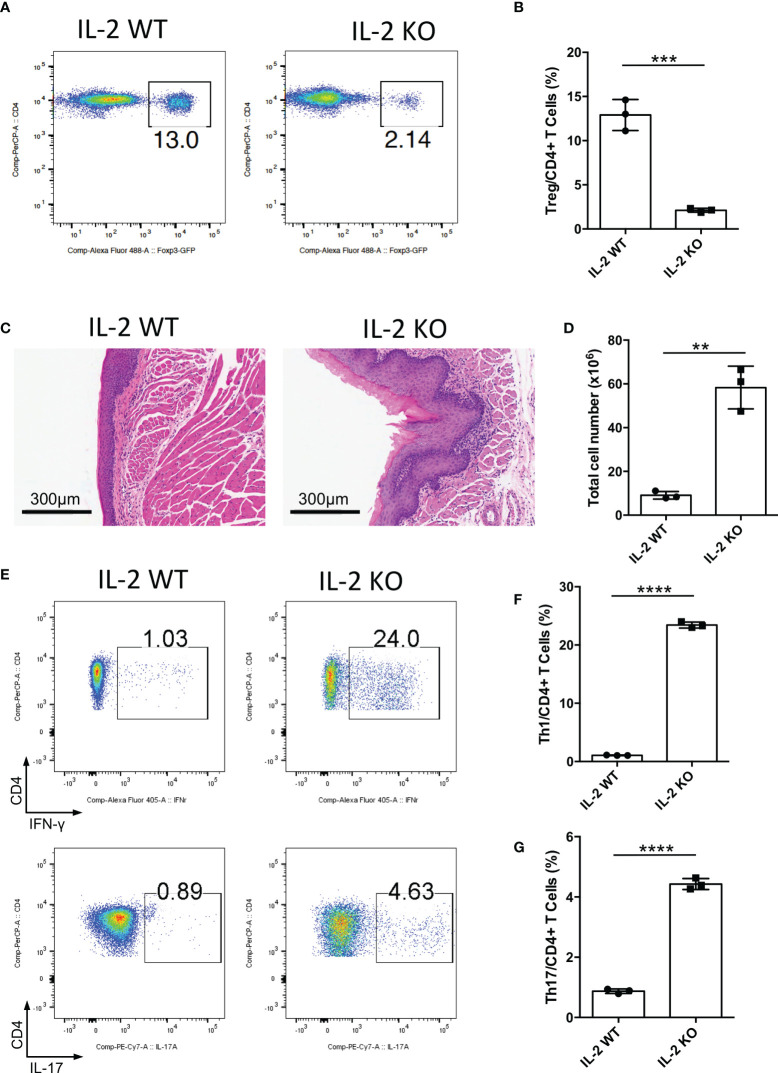
Deletion of IL-2 causes severe inflammation in oral mucosa. Oral tissues and draining lymph nodes (DLNs) of *Il2* ^+/+^ (IL-2 WT) and *Il2* ^-/-^ (IL-2 KO) mice were harvested from four-week-old mice (n=3). **(A, B)** Representative flow cytometry plots **(A)** and bar graphs **(B)** showing frequencies of CD4^+^ Foxp3^+^ Treg cells in DLNs of the oral cavity. **(C)** Representative histology images of oral mucosa sections. Scale bars, 300μm. **(D)** Bar graphs showing total cell numbers in DLNs of the oral cavity. **(E-G)** Representative flow cytometry plots **(E)** and bar graphs **(F, G)** showing frequencies of CD4^+^ IFN-γ^+^ T cells (Th1 cells) and CD4^+^ IL-17A^+^ T cells (Th17 cells) in DLNs. Data are representative of two independent experiments. Summary data are presented as mean ± SD. **p < 0.01, ***p < 0.001, ****p < 0.0001, unpaired two-tailed Student’s t tests.

IL-2 is a vital cytokine for Treg cell development, and the loss of IL-2 causes the deficiency of mature Treg cells (20–22). To further confirm the function of Treg cells in the oral mucosa, we bred *Il2* ^-/-^ mice to investigate inflammation development of oral mucosa in these mice. We confirmed that Treg cell generation in *Il2* ^-/-^ mice was indeed impaired in DLNs of the oral mucosa (**Figures 2A, B**). The histological slides of the oral mucosa of *Il2* ^+/+^ and *Il2* ^-/-^ mice revealed that loss of IL-2 caused severe inflammation in the oral mucosa (**Figure 2C**). Consistent with this, the total cell number of DLNs in *Il2* ^-/-^ mice was significantly increased (**Figure 2D**), and the frequencies of CD4+ and CD8+ T cells did not change significantly (**Supplementary Figures 2A, B**). To check the immune responses in the oral cavity, we investigated the frequencies of Th1 cells and Th17 cells in DLNs, and we found that the deletion of IL-2 caused a dramatic increase in Th1 cells and Th17 cells (**Figures 2E–G**). These data show that deficiency of the IL-2-CD25 signaling pathway indeed causes inflammation in the oral mucosa, further confirming that Treg cells are important in maintaining the immune homeostasis in the oral mucosa.

### Adoptive transfer of CD4^+^CD25^-^CD45Rb^high^ T cells into *Rag1* ^-/-^ mice induces oral mucosal inflammation

**Figure 3:**
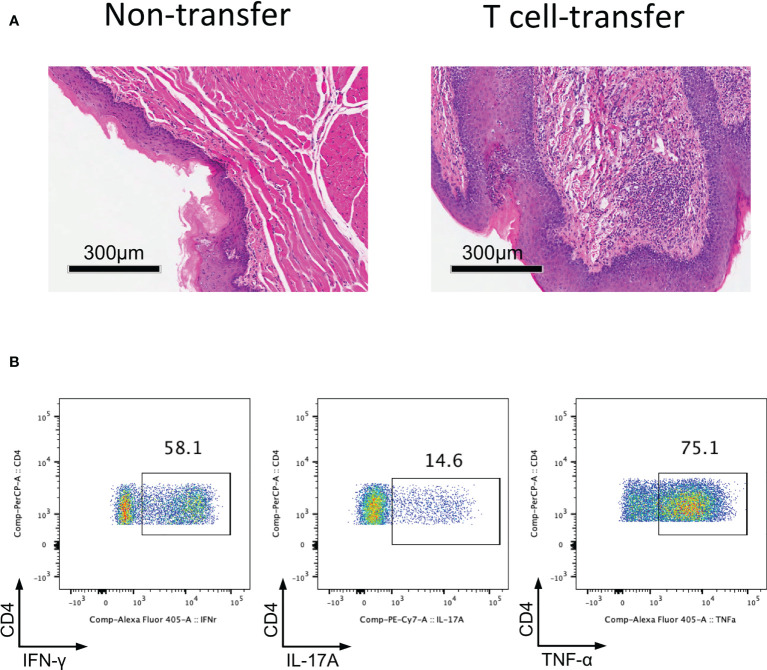
Adoptively transfer CD4^+^CD25^-^CD45Rb^high^ T cells into Rag1^-/-^ mice induces oral mucosal inflammation. CD4^+^CD25^-^CD45RB^high^ T cells sorted by flow cytometry from spleens and peripheral lymph nodes (PLNs) of CD45.1 mice were injected into *Rag1^-/-^* mice, and oral mucosa of *Rag1^-/-^* mice was harvested 8-10 weeks post T cell transfer (n=3). **(A)** Representative histology images of oral mucosa sections. **(B)** Representative flow cytometry plots showing frequencies of Th1 cells, Th17 cells and CD4^+^TNF-α^+^ cells in DLNs of the oral cavity in T cell transfer mice. Data are representative of two independent experiments.

Since we determined that impaired Treg cell-mediated immune homeostasis led to inflammation of the oral mucosa; therefore, we surmise that effector T cells will cause inflammation of oral mucosa in the absence of functional Treg cells. To investigate this hypothesis, we transferred CD4^+^CD25^-^CD45Rb^high^ T cells (naïve T cells) into *Rag1* ^-/-^ mice and allowing the cells to develop into effector T cells. Severe inflammation developed in the oral mucosa of these mice 8-10 weeks after T cell transfer (**Figure 3A**). To identify the immune responses of oral mucosa in these mice, we investigated the immune responses of T cells in DLNs with FCM. We found that plenty of Th1 cells and Th17 cells were present in DLNs of the oral cavity (**Figure 3B**), suggesting that the development of the inflammation is due to the activation of naïve T cells and the proliferation and differentiation of effector T cells in the absence of Treg cells. These data show that the CD4^+^CD25^-^CD45Rb^high^ T cell adoptive transfer model is a good disease model for investigating inflammation of the oral mucosa, as the characteristics of the immune response in this mouse model are very similar to those in patients with oral inflammation (6).

### Adoptive transfer of Treg cells suppresses inflammation in oral mucosa

**Figure 4:**
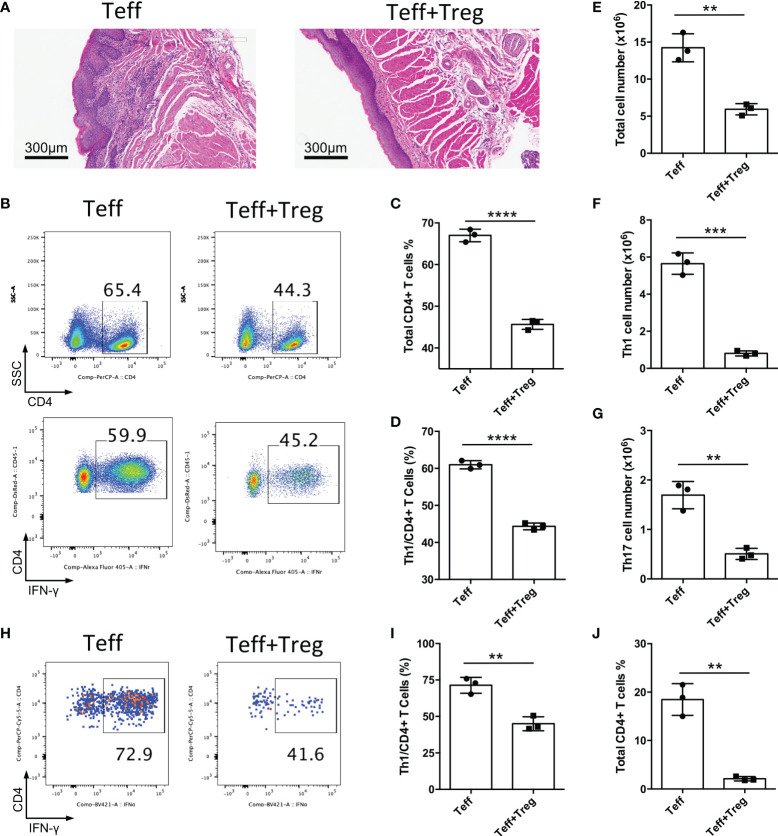
Adoptive transfer of Treg cells suppresses inflammation in oral mucosa. CD4^+^CD25^-^CD45RB^high^ T cells sorted by flow cytometry from the spleens and peripheral lymph nodes (PLNs) of CD45.1 mice were injected into *Rag1^-/-^* mice (Teff), and CD4^+^CD25^+^Foxp3(eGFP)^+^ Treg cells of CD45.2 mice were co-transferred to treat the oral inflammation (Teff+Treg) (n=3). **(A)** Representative histology images of oral mucosa sections. **(B-D)** Representative flow cytometry plots **(B)** and bar graphs showing frequencies of CD45.1^+^ Teff cells **(C)** and Th1 cells **(D)** in DLNs of the oral cavity. **(E-G)** Bar graphs showing the numbers of total immune cells **(E)**, Th1 cells **(F)** and Th17 cells **(G)** in DLNs of the oral cavity. **(H-J)** Representative flow cytometry plots **(H)** and bar graphs showing frequencies of Th1 cells **(I)** and total Teff cells **(J)** in the oral mucosa. Data are representative of two independent experiments. Summary data are presented as mean ± SD. **p < 0.01, ***p < 0.001, ****p < 0.0001, unpaired two-tailed Student’s t tests.

The CD4^+^CD25^-^CD45Rb^high^ T cell adoptive transfer model could be a disease model of oral mucosal inflammation; therefore, we used this model to identify whether adoptive transfer of functional Treg cells could suppress chronic inflammation of the oral mucosa. CD4^+^CD25^-^CD45Rb^high^ T cells isolated from CD45.1^+^ mice were transferred into *Rag1* ^-/-^ mice to induce oral inflammation, with or without the co-transfer of CD4^+^CD25^+^Foxp3(eGFP)^+^ Treg cells sorted from Foxp3-eGFP mice. We found that the adoptive transfer of Treg cells suppressed the development of oral mucosal inflammation; the control group (Teff) developed oral mucosa inflammation, whereas the Treg cell-treated group (Teff+Treg) did not 8-10 weeks after T cell transfer (**Figure 4A**). The changes in immune responses were determined after the mice were euthanized. Treg cell transfer significantly reduced both whole CD4^+^ T cell frequency and Th1 cell frequency in DLNs (**Figures 4B–D**). Moreover, the numbers of total immune cells, Th1 cells and Th17 cells in DLNs were also reduced in the Treg cell-treated group (**Figures 4E–G**). To further confirm that Treg cell therapy can suppress immune responses in the oral mucosa, the immune responses in the oral mucosa were determined by FCM. We found that Treg cell transfer significantly reduced both whole CD4^+^ T and Th1 cell frequencies in the oral mucosa (**Figures 4H–J**). Taken together, these data show that the adoptive transfer of Treg cells can suppress the development of inflammation in the oral mucosa.

## Discussion

In this study, Treg cell deficiency was shown to cause oral mucosal inflammation in mice. Next, the adoptive transfer of CD4^+^CD25^-^CD45Rb^high^ T cells into *Rag1* ^-/-^ mice was shown to induce oral mucosal inflammation. By using this model of oral mucosal inflammation, we confirmed that Treg cell therapy indeed can suppress inflammation of oral mucosa. Therefore, we showed that the CD4^+^CD25^-^CD45Rb^high^ T cell adoptive transfer model, also called the adoptive transfer inflammatory bowel disease (IBD) model, is a good disease model for investigating inflammation of the oral mucosa. Consistent with Treg cell therapy in IBD model (18, 28), the adaptive transfer of Treg cells could also suppress inflammation of the oral mucosa.

Here, we determined that the T cell transfer mouse model of chronic inflammation could be used as a disease model of oral mucosal inflammation, as the immune responses in the oral mucosa of this mouse model are very similar to those in patients with oral inflammation (6, 10, 29, 30). Although the adoptive transfer of CD4^+^CD25^-^CD45Rb^high^ T cells could result in oral mucosal inflammation, this disease model may develop systemic inflammation. Therefore, this disease model is not an ideal disease model for investigating the immune responses of the oral mucosa. Thus, the development of better models of oral inflammatory diseases remains an important bottleneck to overcome in future studies.

The integrity of the oral mucosa is crucial for defending against foreign antigens and maintaining homeostasis of the oral cavity (31). Treg cells have long been considered to be effective in controlling oral mucosa inflammation; however, no study has ever confirmed it strongly. One study reported that the depletion of Treg cells causes the infiltration of effector T cells that are associated with inflammation of the oral mucosa (31). In the current study, we demonstrated that adoptive transfer of Treg cells could suppress oral mucosal inflammation. Together, these two studies show that Treg cell therapy is a promising novel strategy for the treatment of oral inflammatory diseases.

In summary, our current study shows that the T cell transfer mouse model is a good model for investigating oral mucosal inflammation. More importantly, this study also verified that the adoptive transfer of Treg cells could suppress oral mucosa inflammation, showing that Treg cell therapy could be a promising novel strategy to treat oral inflammatory diseases.

## Data availability statement

The original contributions presented in the study are included in the article/**Supplementary Material**. Further inquiries can be directed to the corresponding authors.

## Ethics statement

The animal study was reviewed and approved by Animal Care and Use Committees of the West China Hospital of Stomatology, Sichuan University.

## Author contributions

NX and YW designed and performed the experiments, analyzed the data and drafted the manuscript. HC, HL, and XF performed the experiments. FZ and QC supervised the study and edited the manuscript. XZ and NJ supervised the study, designed the experiments and wrote the manuscript. All authors contributed to the article and approved the submitted version.

## Funding

This work was supported by the National Natural Science Foundation of China (NO. 81991502, U19A2005, 82270986, 81600876), the CAMS Innovation Fund for Medical Sciences (CIFMS, 2019-I2M-5-004, 2020-I2M-C&T-A-023) and the Key Project of the Science and Technology Department of Sichuan Province (NO. 2022YFS0003, 2020YFS0210).

## Conflict of interest

The authors declare that the research was conducted in the absence of any commercial or financial relationships that could be construed as a potential conflict of interest.

## Publisher’s note

All claims expressed in this article are solely those of the authors and do not necessarily represent those of their affiliated organizations, or those of the publisher, the editors and the reviewers. Any product that may be evaluated in this article, or claim that may be made by its manufacturer, is not guaranteed or endorsed by the publisher.
