# Supplementary material for: Regulatory T cell therapy suppresses inflammation of oral mucosa
Source: Front Immunol. 2022 Oct 31;13:1009742. doi: 10.3389/fimmu.2022.1009742 (PMC9660253; doi:10.3389/fimmu.2022.1009742)
Supplement: Supplementary file 1 [file DataSheet_1.pdf]

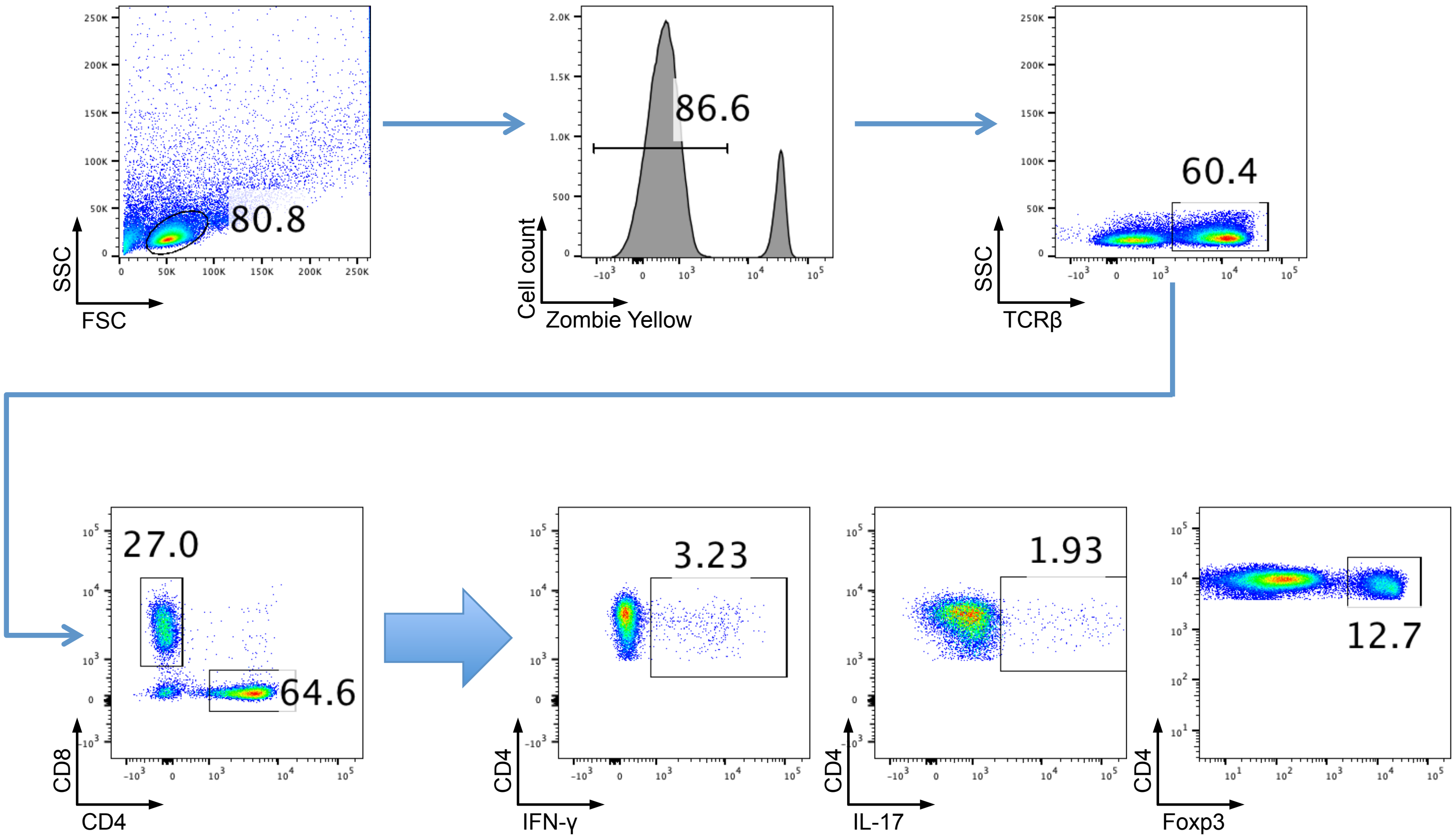

Supplementary Figure 1. Gating strategies for flow data. Samples were stained with Zombie Yellow to separate live cells and dead cells. Then, TCRβ, CD4, CD8, IFN-γ, IL-17 and Foxp3 antibodies were used to stain cells to investigate the frequencies of $CD4^+$ IFN-γ^+ T cells (Th1 cells), $CD4^+$ IL-$17A^+$ T cells (Th17 cells) and $CD4^+$ $Foxp3^+$ T cells (Treg cells).

Figure S1

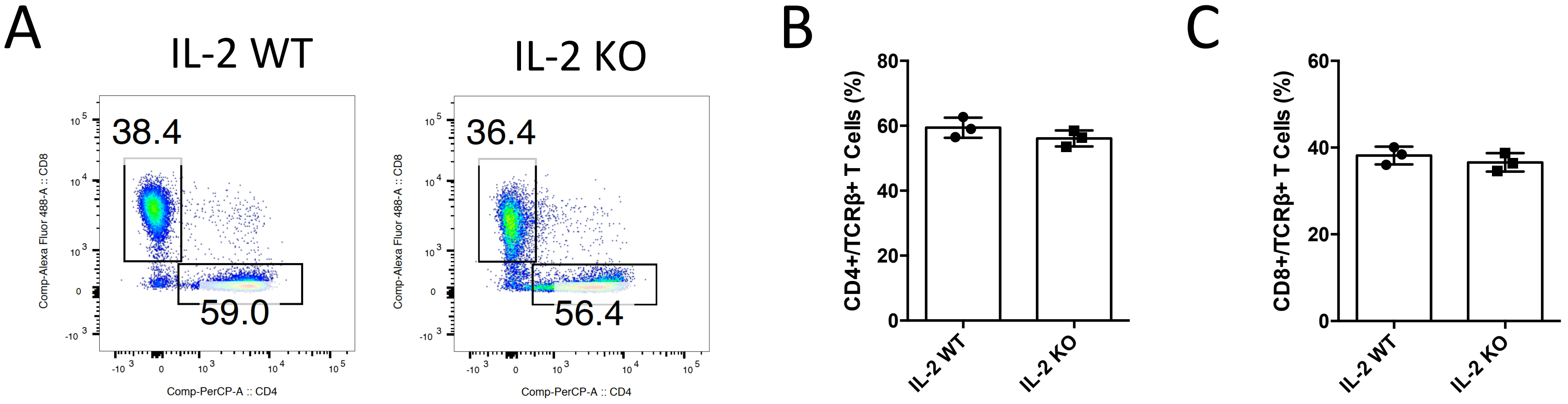

Supplementary Figure 2. Deletion of IL-2 does not change the frequencies of CD4$^+$ and CD8$^+$ T cells in DLNs of the oral cavity. Draining lymph nodes (DLNs) of *Il2*$^{+/+}$ (IL-2 WT) and *Il2*$^{-/-}$ (IL-2 KO) mice were harvested from four-week-old mice (n=3). (**A-C**) Representative flow cytometry plots (**A**) and bar graphs (**B, C**) showing frequencies of CD4$^+$ and CD8$^+$ T cells in DLNs. Data are representative of two independent experiments. Summary data are presented as mean ± SD.

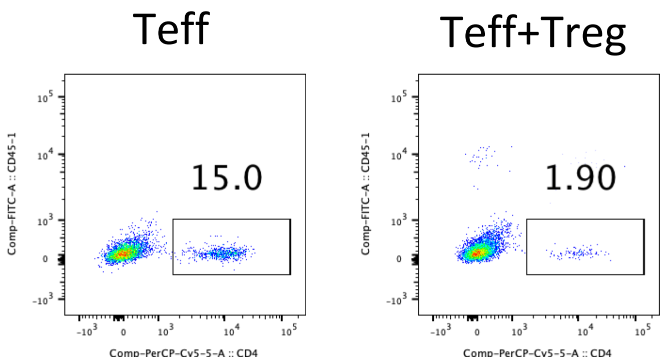

Supplementary Figure 3. Adoptive transfer of Treg cells suppresses inflammation in oral mucosa. $CD4^{+}CD25^{-}CD45RB^{hi}$ T cells sorted by flow cytometry from the spleens and peripheral lymph nodes (PLNs) of CD45.1 mice were injected into *Rag1*$^{-/-}$ mice (Teff), and $CD4^{+}CD25^{+}Foxp3(eGFP)^{+}$ Treg cells of CD45.2 mice were co-transferred to treat the oral inflammation (Teff+Treg). Representative flow cytometry plots show that Treg cell transfer reduced whole CD4+ T cell frequency in oral mucosa.

Figure S3
